# Retrospective Analysis of the Incidence of Drug-Induced Interstitial Lung Disease by Epidermal Growth Factor Receptor Tyrosine Kinase Inhibitors and Survival in Patients Aged 75 Years or Older with Lung Cancer

**DOI:** 10.31662/jmaj.2022-0211

**Published:** 2023-04-10

**Authors:** Masayuki Ishibashi, Yoshiko Nakagawa, Tetsuo Shimizu, Yasuhiro Gon, Hiroshi Yamamoto

**Affiliations:** 1 Division of Respiratory Medicine, Tokyo Metropolitan Institute for Geriatrics and Gerontology, Tokyo, Japan; 2 Division of Respiratory Medicine, Department of Internal Medicine, Nihon University School of Medicine, Tokyo, Japan

**Keywords:** epidermal growth factor receptor tyrosine kinase inhibitors, older patients, lung cancer, osimertinib

## Abstract

**Introduction:**

To date, the appropriate epidermal growth factor receptor tyrosine kinase inhibitors (EGFR-TKIs) for patients aged ≥75 years with advanced *EGFR* mutation-positive, nonsmall cell lung cancer remain unknown.

**Methods:**

This study included a total of 89 patients aged ≥75 years who were diagnosed with *EGFR* mutation-positive, nonsmall cell lung cancer and treated with EGFR-TKIs at the Tokyo Metropolitan Geriatric Hospital and Nihon University ITABASHI Hospital from 2009 to 2020. The patients were classified into five groups based on their treatment: gefitinib (n = 23), erlotinib (n = 4), afatinib (n = 3), first-line osimertinib (n = 23), and TKI to TKI (n = 36). The efficacy and safety of each EGFR-TKI were analyzed.

**Results:**

No significant differences in the overall survival and progression-free survival were observed among the groups. However, a significantly higher incidence of drug-induced interstitial lung disease (ILD) was detected with osimertinib than with the first-generation EGFR-TKIs (p = 0.008).

**Conclusions:**

In older patients with *EGFR* mutation-positive lung cancer, the incidence of drug-induced ILD was significantly increased during osimertinib treatment. This outcome should be noted when treating older patients with osimertinib who may not always want to live longer but want to live better.

## Introduction

The standard of care for patients with advanced epidermal growth factor receptor (EGFR) mutation-positive, nonsmall cell lung cancer is EGFR tyrosine kinase inhibitors (EGFR-TKIs). There are multiple generations of EGFR-TKIs. In Japan, the first-, second-, and third-generation EGFR-TKIs (gefitinib and erlotinib, afatinib and dacomitinib, and osimertinib, respectively) were available in 2022. In the FLAURA study, osimertinib was found to be superior to gefitinib and erlotinib and to prolong progression-free survival. However, only a small percentage (14.2%) of patients aged ≥75 years participated in that study ^(1)^. The NEJ003 and JO22903 studies have suggested that the efficacy and safety of gefitinib and erlotinib in older patients are the same as in younger patients ^(2), (3)^; however, only a few clinical trials have reported a high level of evidence for this suggestion. For this reason, the Japan Lung Cancer Society guidelines for lung cancer treatment recommend osimertinib for use as the primary treatment for patients with advanced nonsmall cell lung cancer, with performance status 0-1 and common mutations of the *EGFR* gene. These guidelines only recommend kinase inhibitors that target genetic mutations in patients aged ≥75 years with no reference to individual EGFR-TKIs. With the increasing numbers of older patients having nonsmall cell lung cancer, the lack of therapeutic evidence for these patients needs to be addressed. This two-center, retrospective study was conducted to determine which EGFR-TKIs could be appropriate for older patients with *EGFR* mutation-positive, nonsmall cell lung cancer based on safety and efficacy.

## Materials and Methods

### Patients

This study included a total of 89 patients aged ≥75 years who were diagnosed with *EGFR* mutation-positive, nonsmall cell lung cancer without interstitial pneumonia and received EGFR-TKIs at the Tokyo Metropolitan Geriatric Hospital and Nihon University ITABASHI Hospital from 2009 to 2020. Patient characteristics, *EGFR* mutation subtypes, treatment profiles, and prognoses were extracted from their medical records.

This study was approved by the Institutional Review Board of Tokyo Metropolitan Geriatric Hospital and Nihon University School of Medicine R22-006). All methods were performed according to the approved guidelines. This was a retrospective, observational study conducted using the opt-out method noted on the institutions’ websites.

### Statistical analysis

Based on the EGFR-TKIs administered, the patients were classified into five groups: gefitinib (n = 23), erlotinib (n = 4), afatinib (n = 3), first-line osimertinib (n = 23), and TKI to TKI (n = 36). The gefitinib, erlotinib, afatinib, and first-line osimertinib groups consisted of patients who received each EGFR-TKI as first-line therapy and no second-line therapy at the time of analysis. On the other hand, the TKI to TKI group consisted of patients who received an EGFR-TKI as first-line therapy and received another EGFR-TKI as second-line or later therapy at the time of analysis. The overall survival and progression-free survival were analyzed using the Kaplan-Meier method and log-rank test. The two-sided Mann-Whitney U test or Fisher’s exact test was used for data analysis. For the progression-free survival, the TKI duration was calculated by combining the gefitinib and erlotinib groups (first-generation TKI group). The osimertinib duration was calculated for all patients in the first-line osimertinib group (first-line osimertinib group). The afatinib duration was calculated for all patients in the afatinib group (afatinib group). For the overall survival, the gefitinib and erlotinib groups were combined with the first-generation TKI group, and the first-line osimertinib and afatinib groups were independent, as was the calculation of the progression-free survival. The progression-free survival and overall survival in the TKI to TKI group were excluded from the analysis as they were considered to suffer from a large bias. On the other hand, safety analysis was included in the TKI to TKI group. The median observation period of the progression-free survival in all three groups was 10.1 months, whereas that of the overall survival was 18.2 months. Patients who were alive at the end of the observation period or whose last date of survival could not be confirmed for any reason were considered “censored.”

p < 0.05 was considered to indicate statistical significance, unless otherwise specified. All statistical analyses were conducted using EZR (Saitama Medical Center of Jichi Medical University, Shimotsuke, Japan). This is a graphical user interface for the R programming language developed by the R Foundation for Statistical Computing. EZR is a modified version of R Commander (Free Access) created by importing statistical functions frequently used in biostatistics into the latter ^(4)^.

## Results

### Patient characteristics (Table 1)

The median age of the patients was 79 years in the gefitinib and erlotinib groups, 77 years in the afatinib and first-line osimertinib groups, and 78 years in the TKI to TKI group. Most patients had good performance status. Almost all patients had unresectable, advanced cancer without an indication for radiation therapy; however, three patients in the gefitinib group received gefitinib instead of undergoing surgery or radiation therapy due to patient choice. All patients had common *EGFR* mutations. Two of the 23 patients in the gefitinib group and 15 of the 36 patients in the TKI to TKI group had a T790M acquisition resistance mutation. For 15 of the 36 patients in the TKI to TKI group, osimertinib was chosen for subsequent treatment. In addition, patients in the TKI to TKI group tended to receive cytotoxic agents during their entire treatment course as opposed to the other groups. One patient receiving osimertinib, five receiving gefitinib, two receiving erlotinib, and one receiving afatinib as their first-line TKI required a dose reduction in the TKI to TKI group. Furthermore, four patients receiving osimertinib, two receiving gefitinib, and six receiving erlotinib as their second-line TKI required a dose reduction in this group.

**Table 1.** Patient Characteristics.

		Gefitinib group	Erlotinib group	Afatinib group	Osimertinib group	TKI to TKI group
N		23	4	3	23	36
First-line TKI		Gefitinib	Erlotinib	Afatinib	Osimertinib	Gefitinib/Erlotinib/ Afatinib/Osimeritinib: 27/5/1/3
Second-line TKI		None	None	None	None	Gefitinib/Erlotinib/ Afatinib/Osimeritinib: 4/15/2/15
Age	Median (range)	79 (75-92)	79 (78-91)	77 (75-78)	77 (75-91)	78 (75-91)
Gender	Male/female	7/16	0/4	0/3	7/16	14/22
Smoking index	Median (range)	0 (0-480)	0 (0)	0 (0)	0 (0-2820)	0 (0-500)
Clinical stage	I/II/III/IV/ recurrence	2/1/3/12/5	0/0/0/2/2	0/0/0/2/1	0/0/2/16/5	0/0/5/25/6
Performance status	0/1/2/3	11/8/2/2	2/1/1/0	2/1/0/0	9/11/3/0	17/19/0/0
Charlson comorbidity index	Median (range)	1 (0-10)	1 (0-1)	2 (1-2)	1 (0-4)	0 (0-3)
EGFR gene mutation	Exon19 deletion/ L858R/Others	12/11/0	1/3/0	3/0/0	11/10/02	19/14/3
*De novo *T790M mutation		0	0	0	1	0
T790M acquisition resistance mutation		2	0	0	0	15
Treatment history of cytotoxic agents	Yes/No	3/20	1/13	2/1	4/19	19/17
Objective response rate of first-line EGFR-TKI		70%	50%	100%	74%	70%
Objective response rate of second-line EGFR-TKI		-	-	-	-	43%
Adverse events other than ILD		Rash (n = 5)	Rash (n = 1)	Diarrhea (n = 3)	Diarrhea (n = 8)	Rash (n = 14)
		Liver dysfunction (n = 3)	Paronychia (n = 1)	Nausea (n = 1)	Rash (n = 8)	Liver dysfunction (n = 5)
		Paronychia (n = 1)			Paronychia (n = 5)	Diarrhea (n = 5)
		Nausea (n = 1)			Nausea (n = 1)	Paronychia (n = 4)
		Sepsis (n = 1)				Nausea (n = 3)
						Leukocytopenia (n = 1)
Dose reduction of first-line EGFR-TKI	Yes/No	3/20	1/3	3/0	5/18	9/27
Dose reduction of second-line EGFR-TKI	Yes/No	-	-	-	-	12/24
Prognosis	Alive/Dead	11/12	2/2	1/2	12/11	13/23

EGFR-TKI, epidermal growth factor receptor tyrosine kinase inhibitor; ILD, interstitial lung disease

### Efficacy (Figure 1)

At the time of analysis, all 33 patients were found to have disease progression, and 27 of the 53 had died. The median progression-free survival was 14.8 months in the first-generation TKI group, 18.2 months in the first-line osimertinib group, and 19.8 months in the afatinib group (p = 0.697). On the other hand, the median overall survival was 55.7 months in the first-generation TKI group, 49.1 months in the first-line osimertinib group, and 96.4 months in the afatinib group (p = 0.719).

**Figure 1.**
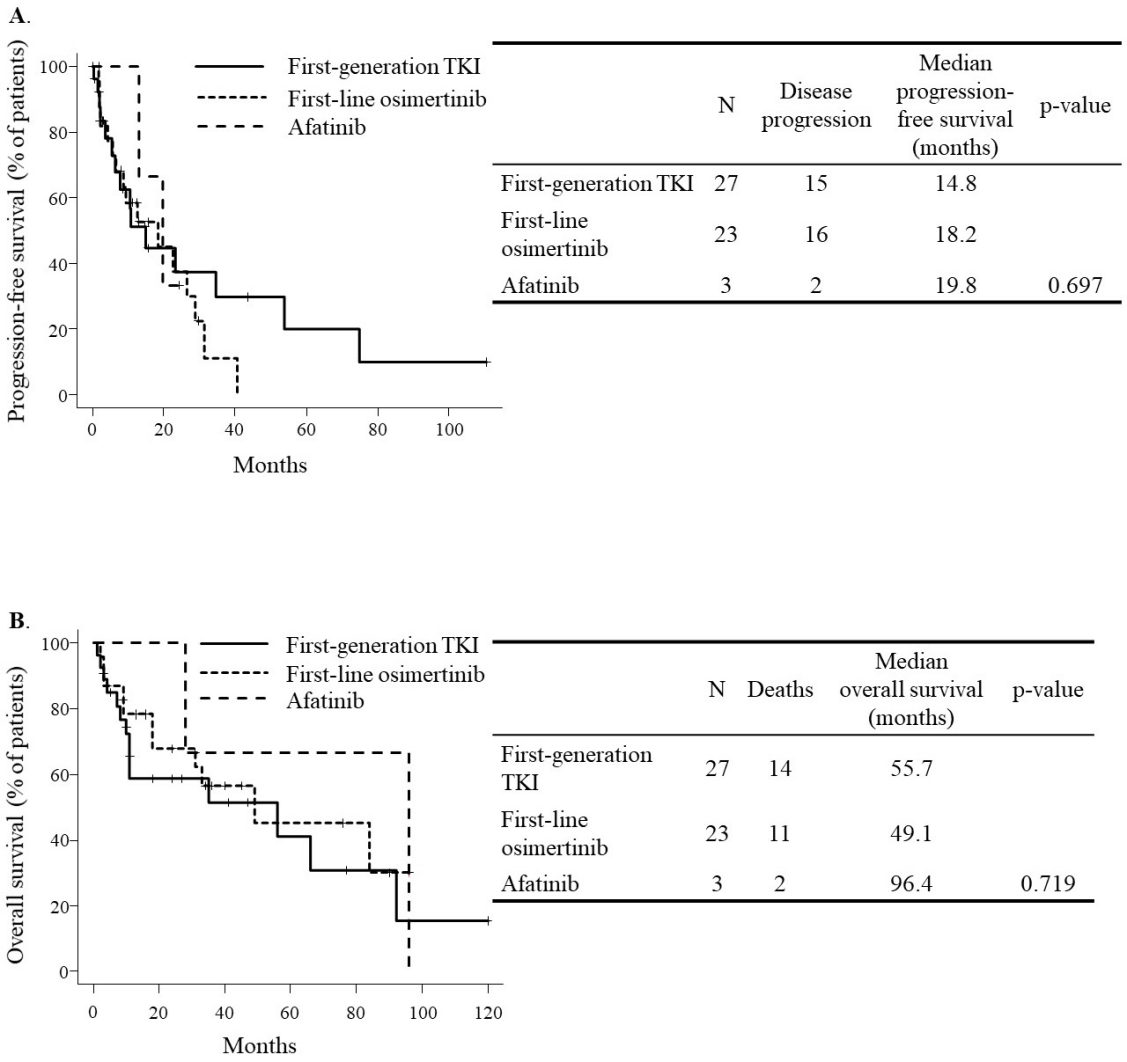
Efficacy of epidermal growth factor receptor tyrosine kinase inhibitors A. Progression-free survival B. Overall survival.

### Safety (Tables 1, 2, and 3)

No adverse events were significantly more common among the groups other than drug-induced interstitial lung disease (ILD) (Table 1). Seven of 41 patients (17%) had an onset of drug-induced ILD during osimertinib treatment, whereas 2 of 78 patients (2%) had an onset during first-generation TKI treatment (p = 0.008) (Table 2). Three patients developed drug-induced ILD while receiving osimertinib in the TKI to TKI group after more than 1 month of treatment. Among the patients who developed drug-induced ILD, an 82-year-old female receiving osimertinib as first-line treatment died, whereas the others recovered either by osimertinib withdrawal or high-dose corticosteroid therapy (Table 3). No patients resumed osimertinib treatment after recovering from drug-induced ILD.

**Table 2.** Occurrence of ILD.

EGFR-TKI causing ILD	N	*P*-value
First- or second-line osimertinib (n = 41)	7	
First-generation TKIs (n = 78)	2	0.008

EGFR-TKI, epidermal growth factor receptor tyrosine kinase inhibitor; ILD, interstitial lung disease

**Table 3.** Patients with Drug-Induced Lung Disease.

Age (years)	Gender	Performance status	Smoking index	EGFR gene mutation	Causative TKI	Duration of administration (days)	Treatment for ILD	Other adverse events (CTCAE version 5)
88	Male	2	480	Exon19 deletion	Gefitinib	327	Withdrawal of TKI	None
88	Male	1	600	Exon19 deletion	Gefitinib	855	Intravenous corticosteroid	None
84	Female	1	0	L858R	First-line osimertinib	242	Intravenous corticosteroid	None
82	Female	0	0	Exon19 deletion	First-line osimertinib	53	Intravenous corticosteroid	None
77	Female	1	0	L858R	First-line osimertinib	285	Intravenous corticosteroid	Skin rash (grade 2)
76	Male	0	2820	Exon19 deletion	First-line osimertinib	58	Withdrawal of TKI	Skin rash (grade 2)
76	Female	1	0	L858R	Second-line osimertinib	430	Withdrawal of TKI	None
87	Male	1	500	L858R	Second-line osimertinib	69	Intravenous corticosteroid	Paronychia (grade 2)
81	Male	1	450	Exon19 deletion	Second-line osimertinib	31	Intravenous corticosteroid	Aspartate aminotransferase increased (grade 1)

EGFR-TKI, epidermal growth factor receptor tyrosine kinase inhibitor; ILD, interstitial lung disease; CTCAE, common terminology criteria for adverse events

## Discussion

This study, where mortality was observed in more than half the patients analyzed, could not replicate the results of the FLAURA study ^(1)^, which could have provided a rationale for selecting osimertinib as first-line therapy for patients aged ≥75 years with *EGFR* mutation-positive, nonsmall cell lung cancer. Patients receiving osimertinib had a significantly higher incidence of drug-induced ILD, and one patient died of drug-induced ILD. Although the overall survival results of this study should not be generalized, considering the possibility of insufficient tracking by shorter observation periods, this is an interesting, real-world data for older patients.

Nakao et al. conducted a single-arm, phase 2 study (SPIRAL study) of osimertinib in 36 older patients with nonsmall cell lung cancer positive for the *EGFR* T790M acquisition resistance mutation as a second or subsequent line of therapy ^(5)^. The median progression-free survival of these patients was 11.9 months, the objective response rate was 58.3%, and 11.1% of the patients reported pneumonitis as an adverse event. The frequency of pneumonitis was lower in the SPIRAL study than that in osimertinib groups in this study. However, in a subset analysis of Japanese patients in the FLAURA study, the incidence of ILD with osimertinib was reported to be 12.3%, which was higher than the 2% rate for first-generation TKIs with gefitinib or erlotinib^(6)^.

Yamamoto et al. also reported a single-arm, retrospective, observational study of 132 patients aged ≥75 years who received osimertinib at 19 hospitals in Japan (HOT2002). According to the results of HOT2002, the incidence of ILDs in Japanese patients was 17.4% and was the cause of osimertinib discontinuation in two-thirds of the cases ^(7)^.

The patient characteristics in this study were in part similar to those of the FLAURA, SPIRAL, HOT2002, and AURA3 studies. Although comparison between the results of this study with actual administration of EGFR-TKIs and the results of those other studies is difficult, it is unclear whether osimertinib should be recommended as first-line treatment for patients aged ≥75 years with *EGFR* mutation-positive, nonsmall cell lung cancer. The results were confusing.

Akishita et al. reported in their large cohort survey that the priority of healthcare outcomes for older patients was not mortality reduction ^(8)^. They recommended that a geriatric assessment on physical performance, emotional state, social or economic status, and presence or absence of caregivers should be conducted to comprehensively understand the environment of older patients and to select a flexible treatment plan that is not limited to only survival prolongation ^(9)^. Given these perspectives, the choice of EGFR-TKIs for older patients should be based not only on survival but also on a lower incidence of pneumonitis than with osimertinib. Choosing first-generation EGFR-TKIs, such as gefitinib or erlotinib, as the treatment of choice may be reasonable, as there is a lower chance of discontinuation due to drug-induced ILD with these EGFR-TKIs. They also have the potential for long-term treatment continuation or subsequent cytotoxic chemotherapy. We believe that gefitinib could be an acceptable EGFR-TKI for the treatment of lung cancer patients aged ≥75 years as part of an individualized therapy based on patient values.

This study has several limitations. First, it is only a retrospective study. Second, the small number of patients makes the interpretation of the results difficult to generalize, especially in terms of efficacy validation.

### Conclusion

In older patients with *EGFR* mutation-positive lung cancer who received EGFR-TKIs, the incidence of drug-induced ILD was significantly increased during osimertinib treatment compared with treatment with other EGFR-TKIs. This outcome should be noted when administering osimertinib to older patients who do not always want to live longer but want to live better.

## Article Information

### Conflicts of Interest

H Yamamoto received research funding from CES Descartes Co. Ltd.

M Ishibashi, Y Nakagawa, T Shimizu, and Y Gon have no conflicts of interest to disclose.

### Acknowledgement

The authors would like to thank Enago (www.enago.jp) for the English language review.

### Author Contributions

All authors were involved in drafting the article or revising it critically for important intellectual content. They have also read and approved the final version of the manuscript. Masayuki Ishibashi and Hiroshi Yamamoto were responsible for the design, analysis, interpretation, and drafting of the manuscript. Yoshiko Nakagawa, Tetsuo Shimizu, and Yasuhiro Gon were responsible for creating the database of Nihon University ITABASHI Hospital and revising the manuscript.

### Approval by Institutional Review Board (IRB)

The study was conducted with the approval of the IRB of Tokyo Metropolitan Geriatric Hospital and Nihon University School of Medicine. The IRB approval number is R22-006. All methods were performed according to the approved guidelines. This was a retrospective, observational study conducted using the opt-out method noted on the two institutions’ websites.
